# Molecular epidemiology and virulence factors of methicillin‐resistant *Staphylococcus aureus* isolated from patients with bacteremia

**DOI:** 10.1002/jcla.23077

**Published:** 2019-11-13

**Authors:** Hyun Jin Kim, Qute Choi, Gye Cheol Kwon, Sun Hoe Koo

**Affiliations:** ^1^ Department of Laboratory Medicine Chungnam National University Hospital Daejeon Korea

**Keywords:** bacteremia, methicillin‐resistant *Staphylococcus aureus*, ST5, ST72, virulence gene

## Abstract

**Background:**

The various virulence factors of methicillin‐resistant *Staphylococcus aureus* bacteremia (MRSAB) are associated with a high mortality rate worldwide. Further studies are warranted to confirm the significant relationship between the strains and virulence genes. Here, we prospectively investigated the molecular characteristics underlying the genotypes and virulence factors of MRSA isolated from patients with bacteremia.

**Methods:**

We collected 59 MRSA isolates from adult patients with bacteremia. Antimicrobial susceptibility results were obtained with the Vitek2 automated system. Genotypes were identified with multi‐locus sequence typing (MLST) and pulse‐field gel electrophoresis (PFGE), and 21 virulence genes were detected with polymerase chain reaction (PCR).

**Results:**

The 59 MRSA isolates mainly comprised ST5 (n = 31, 52.5%) and ST72 (n = 22, 37.2%). Most ST5 isolates and all ST72 isolates were clustered into one and two PFGE groups, respectively. The mean number of virulence genes was higher in ST5 than in ST72. *Sel* was more frequently detected in ST5 than in ST72, whereas *sec* and *sed* were found only in ST5. ST5 had significantly higher resistance against many antibiotics than ST72.

**Conclusion:**

Most MRSA isolates causing bacteremia were ST5 (CC5) and ST72 (CC8), and those belonging to the same STs were divided into only a few PFGE groups. ST5 was associated with higher antibiotic resistance and staphylococcal superantigen toxin genes, than ST72, which may be related to its higher virulence.

## 1 INTRODUCTION

Methicillin‐resistant *Staphylococcus aureus* (MRSA) infection is a major concern, owing to the high incidence rate worldwide. Many European Union countries have reported MRSA incidence rates above 25%.^1^ In United States, MRSA accounts for up to 53% of *S aureus*.^2^ Some Asian countries have the highest prevalence of MRSA in the world.^3^ In particular, South Korea has a high MRSA prevalence rate of 60.9% among *S aureus* as per the 2015 annual report of Korean Antimicrobial Resistance Monitoring System (KARMS).^4^

Methicillin‐resistant *Staphylococcus aureus* causes various diseases such as skin and soft tissue infections, endocarditis, and bone and joint infections.^5^, ^6^ Among these infections, MRSA bacteremia (MRSAB) is one of the most common problems because of the associated high mortality rate. The mortality rate for patients with MRSAB is about 30%‐40%,^7^, ^8^, ^9^ which is about twice that reported for methicillin susceptible *S aureus* bacteremia (MSSAB).^10^, ^11^, ^12^, ^13^

Methicillin‐resistant *Staphylococcus aureus* is known to produce various virulence factors, including staphylococcal enterotoxins (SEs), toxic shock syndrome toxin‐1 (TSST‐1), leukocidins, hemolysins, and exfoliative toxins, and immune‐modulatory factors.^14^ Previous studies have reported the association between the virulence factors and mortality rate in patients with MRSAB. Masayuki et al reported the independent association between superantigenic toxins (SAgT) such as TSST‐1 and SEs and 30‐day mortality in MRSAB.^14^ Furthermore, Park et al reported that three staphylococcal superantigen genes (*sec*, *sel*, and *tst*) causing bloodstream infection were associated with mortality.^15^

The virulence genes harbored by each clone have been previously reported,^15^, ^16^, ^17^ and studies have been performed to investigate whether the differences in the virulence genes among clones have any impact on mortality. Park et al reported that the virulence genes possessed by a particular clone were related to mortality.^15^ However, further studies are warranted to investigate the significant relationship between the specific clones associated with MRSAB and their virulence genes.

In the present study, we prospectively investigated the molecular characteristics underlying genotypes and virulence factors of MRSA isolated from patients with bacteremia.

## 2 MATERIALS AND METHODS

### 2.1 Study design

We conducted this prospective cohort study at the Chungnam National University Hospital, which is a 1300‐bed tertiary teaching hospital in Daejeon, South Korea. Adult patients (18 years and older) with MRSAB were included in this study. A total of 59 non‐duplicate MRSA isolates from blood cultures were collected from October 2016 to August 2018. The disk diffusion test for cefoxitin was performed for all MRSA isolates identified by the automated system Vitek2 (bioMérieux), and the results were confirmed according to Clinical and Laboratory Standards Institute (CLSI) guideline.^18^ *mecA* genes were detected using polymerase chain reaction (PCR).^19^

The infection was considered community‐associated (CA) in the following cases: hospitalization <48 hours before positive culture of MRSA; no history of prior hospitalization, residence in a long‐term care facility, surgery within 1 year of MRSA‐positive culture, dialysis within the past year, or previous MRSA infection or colonization; patients without an indwelling catheter or percutaneous device.^20^ Healthcare‐associated (HA) infections included those that did not meet these criteria.^21^

This study was approved by the Institutional Ethics Review Board of Chungnam National University Hospital (IRB No. 2018‐05‐040). No informed consent was acquired because all isolates were generated and analyzed as a part of microbiological diagnostics and therapeutic purpose.

### 2.2 Antimicrobial susceptibility testing

The results of ciprofloxacin, clindamycin, trimethoprim‐sulfamethoxazole (TMP‐SMX), quinupristin‐dalfopristin (Q‐D), erythromycin, fusidic acid, gentamicin, mupirocin, nitrofurantoin, penicillin, G‐D, rifampicin, tetracycline, tigecycline, and linezolid were obtained with the automated system Vitek2 (bioMérieux). The minimal inhibitory concentration (MIC) of vancomycin was determined using the vancomycin *E* test (AB Biodisk) on Mueller‐Hilton agar. All the results of antimicrobial susceptibility were interpreted according to CLSI guideline.^18^

### 2.3 Multi‐locus sequence typing (MLST)

Multi‐locus sequence typing was conducted for all isolates as previously described.^22^ The sequence types (STs) for each isolate were determined by comparing the sequence of each locus with the reference sequence in the *S aureus* MLST database (https://pubmlst.org). Through eBURST, the isolates with similar STs that shared identical alleles at more than 6 of the 7 loci were grouped into a clonal complex (CC) and the evolutionary origin of strains was determined from the primary founder in each CC. Primary founder was assigned to the ST that had the largest number of single‐locus variants (SLVs) in the group. Subgroup founder was defined as a diversified SLV of the primary founder. Singleton was the ST that did not correspond to any clonal group.^1^, ^23^

### 2.4 Pulse‐field gel electrophoresis (PFGE)

Pulse‐field gel electrophoresis was performed for the analysis of the genetic similarity between all MRSA isolates according to the guidelines of the Korea Centers for Disease Control and Prevention (KCDC). In brief, the chromosomal DNA of MRSA was prepared in agarose plugs and cleaved with 50 U *Sma*I enzyme. The samples were subjected to electrophoresis on a 1% agarose gel in 0.5% Tris‐Borate‐EDTA buffer at 14°C using CHEF DR‐III (Bio‐Rad). The switch time included an initial time of 5.2 seconds, final time of 40.2 seconds, and run time of 9 hours at a voltage of 6 V/cm.

Cluster analyses were performed using BioNumerics 7.6 (Applied Math) with dice correlation for band matching at a 1.5% position tolerance and the unweighted pair group method with an arithmetic average (UPGMA) and similarity coefficient of 80%.^1^

### 2.5 Detection of virulence genes

We selected a list of virulence genes based on their prior association with MRSAB. To identify the presence of virulence factors, PCR was performed. The presence of superantigens was examined with multiplex PCR, as previously described.^24^, ^25^, ^26^, ^27^, ^28^, ^29^, ^30^, ^31^ For multiplex PCR, four sets (Set 1; *sea*, *seb*, *sec*, *sed*, *see*, and *femB*; Set 2; *seg*, *seh*, *sei*, *sej*, *sep*, and *femA*; Set 3; *sek*, *sem*, *seo*, and *fem*A; Set 4; *sen*, *sel*, and *femB*) of primer master mixes were prepared, and the PCR was performed with AccuPower^R^ Multiplex PCR PreMix (Bioneer) according to the manufacturer's instructions. Uniplex PCR (*lukDE*, *hlg*, *lukS/F‐PV*, *fnbA*, *sdrD*, and *sdrE*) was carried out with AccuPower^R^ HotStart PCR Premix (Bioneer). PCR products were analyzed using QIAxcel Advanced System, an automated capillary electrophoresis device (Qiagen).

### 2.6 Statistical analysis

To compare the characteristics of ST5 and ST72, analyses were performed using the Fisher exact test. Odds ratios (ORs) and 95% confidence intervals (CIs) were calculated using the logistic regression model and a two‐tailed *P* value < .05 was considered statistically significant. Analyses were performed using SPSS version 21.0 (SPSS Inc).

## 3 RESULTS

A total 59 MRSA isolates were collected. The mean (± standard deviation [SD]) age was 70.2 (±11.2) years. A total of 38 (64.4%) isolates were obtained from males. Among the total MRSA isolates derived from blood cultures, 51 (89.5%) and 8 (10.5%) strains were HA and CA, respectively.

The antibiograms are listed in Table 1. MRSA isolates showed a high resistance to penicillin (98.3%), erythromycin (71.2%), ciprofloxacin (67.8%), tetracycline (55.9%), clindamycin (52.5%), fusidic acid (52.5%), and gentamycin (44.1%).

**Table 1:** The antibiograms of MRSA isolates

Antibiotic	MRSA n = 59 (%)		
	R	I	S
Ciprofloxacin	40 (67.8)	2 (3.4)	17 (28.8)
Clindamycin	31 (52.5)	0 (0)	27 (45.8)
TMP‐SMX	0 (0)	0 (0)	59 (100)
Erythromycin	42 (71.2)	1 (1.7)	16 (27.1)
Fusidic acid	31 (52.5)	2 (3.4)	26 (44.1)
Gentamicin	26 (44.1)	0 (0)	33 (55.9)
Mupirocin	7 (11.9)	15 (25.4)	37 (62.7)
Nitrofurantoin	0 (0)	0 (0)	59 (100)
Penicillin	58 (98.3)	0 (0)	1 (1.7)
Q‐D	0 (0)	0 (0)	59 (100)
Rifampicin	10 (16.9)	0 (0)	49 (83.1)
Tetracycline	33 (55.9)	0 (0)	26 (44.1)
Vancomycin	0 (0)	0 (0)	59 (100)
Tigecycline	0 (0)	0 (0)	54 (91.5)
Linezolid	0 (0)	0 (0)	59 (100)

Abbreviations: I, intermediate; MRSA, methicillin‐resistant *Staphylococcus aureus*; Q‐D, Quinupristin‐dalfopristin; R, resistant; S, sensitive; TMP‐SMX, Trimethoprim‐sulfamethoxazole.

According to the results of vancomycin E tests, all 59 MRSA isolates were sensitive to vancomycin and 27 (45.8%), 26 (44.1%), and 6 (10.2%) out of 59 isolates had a vancomycin MIC ≤ 1.0 µg/mL, 1.0 µg/mL < MIC ≤ 1.5 µg/mL, and MIC > 1.5 µg/mL, respectively.

Virulence genes *seg*, *sei*, *sem*, *sen*, *seo*, *lukDE*, *sdrD*, and *sdrE* were detected in most MRSA isolates (89.5%‐100%), and *sec* and *sel* were observed in about half of MRSA isolates (38.6% and 57.9%, respectively); other genes were rarely recovered (0%‐12.3%)**.**

### 3.1 Molecular epidemiology

**Figure 1:**
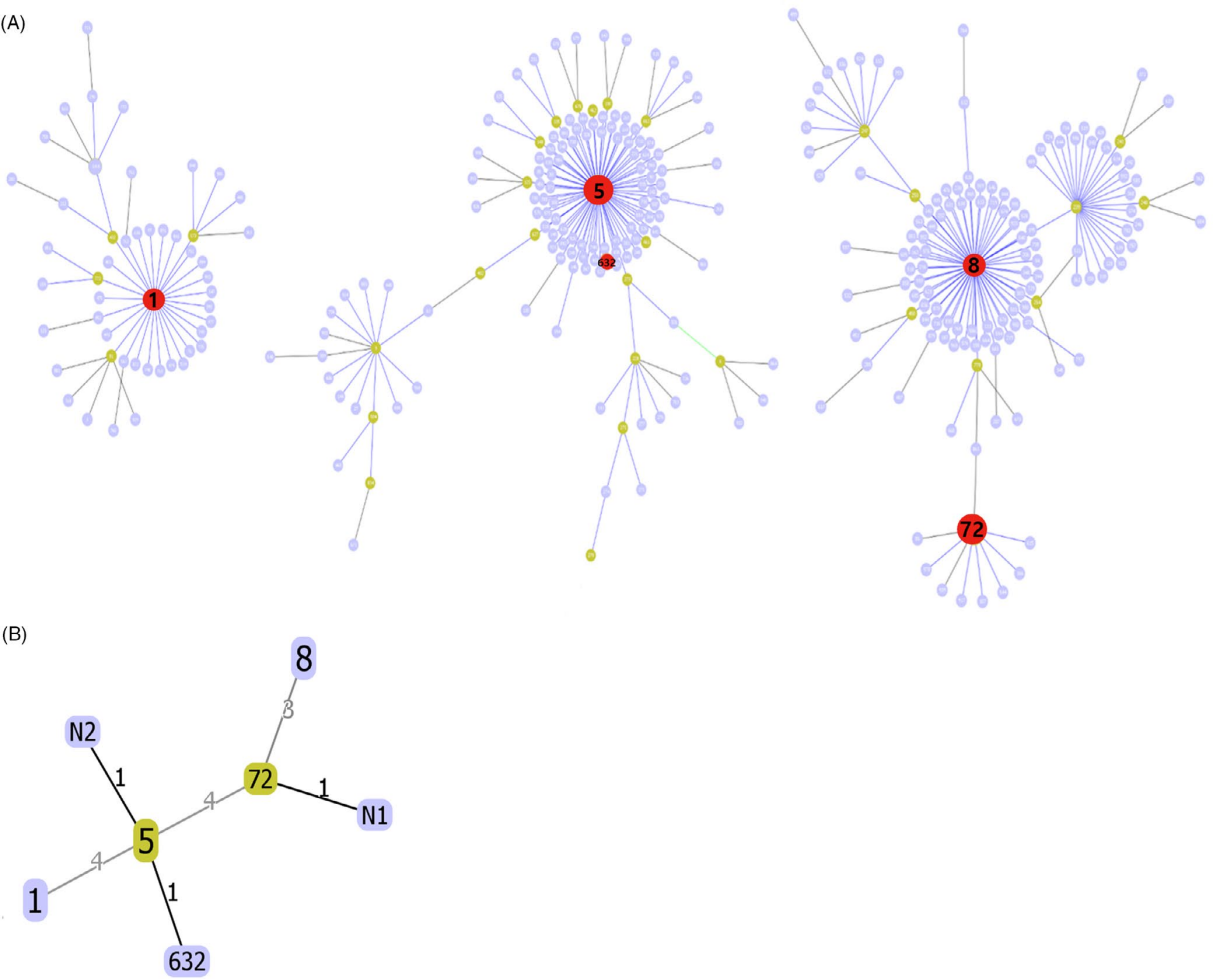
A, Population snapshot of MRSA strains in the MLST database. STs in red color are those found in this study. B, The relationship of STs found in this study. The differences of locus between STs are represented. N1, Novel1; N2, Novel2

Most MRSA isolates comprised ST5 (CC5) (n = 31, 52.5%) and ST72 (CC8) (n = 22, 37.3%). Two isolates (3%) were ST632 (CC5), and one isolate each (2%) was detected as ST1 (CC1) and ST8 (CC8), respectively. ST5 and ST8 were the primary founders of CC5 and CC8, respectively. ST72 was the trilocus variant (TLV) of ST8, belonged to CC8. ST632 and Novel2 were SLVs of ST5 that belonged to CC5. Novel1 belonged to CC8 (Figure 1A and B).

**Figure 2:**
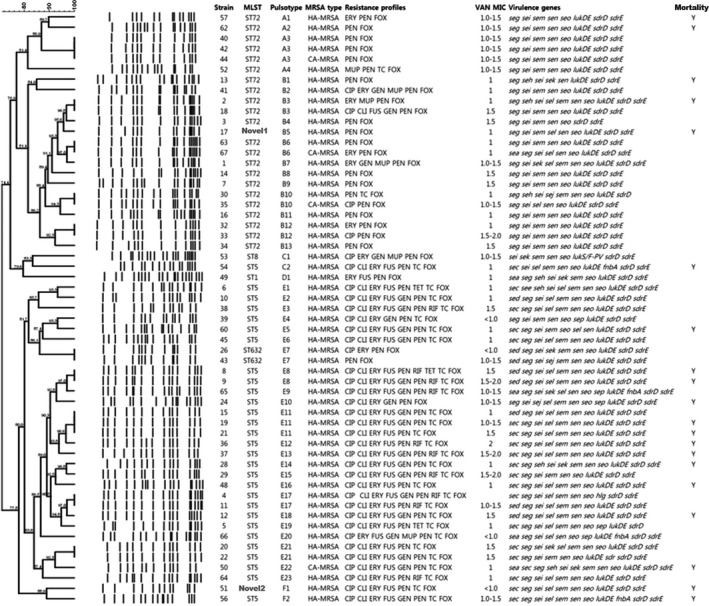
Dendrogram of PFGE patterns for MRSA isolates generated by UPGMA clustering method using Dice coefficient

PFGE results of 57 MRSA isolates (results of two isolates were excluded owing to the absence of a clear banding pattern) were differentiated into 45 pulsotypes (A1‐F2). Based on 80% similarity, six PFGE groups were detected. One PFGE group (D) comprised only a single pulsotype. Group E was the predominant PFGE group (n = 29; 51%) with multiple pulsotypes (E1‐E23), followed by group B (n = 17, 30%) with pulsotype B1 to B13 and group A (n = 6, 11%) with pulsotype A1‐A4. All ST5 were clustered into PFGE group E except for two isolates. ST72 were divided into PFGE group A and B (Figure 2).

### 3.2 Phenotypic and molecular characteristics

We observed differences in the antibiotic susceptibility test results and virulence genes between genotypes (STs), especially ST5 and ST72, which were the two major clones isolated from patients with MRSAB. The results are shown in Table 2. ST5 had a higher resistance to antibiotics than ST72 (ST5: 8.0 ± 0.8, ST72: 3.0 ± 1.3, respectively, *P* < .001). ST5 showed significantly higher resistance rates against many antibiotics, including clindamycin, erythromycin, fusidic acid, gentamicin, rifampicin, and tetracycline, than ST72. However, no significant difference was observed between the susceptibility of ST5 and ST72 to vancomycin.

**Table 2:** The results of the antibiotic resistance and virulence genes according to ST5 and ST72

Variable	ST5 n = 31 (%)	ST72 n = 22 (%)	*P* value	OR (95% CI)
Resistance of antibiotics				
N. of resistant antibiotics (mean ± SD)	8.0 ± 0.8	3.0 ± 1.3	**<.001**	
Vancomycin MIC ≥ 1.5 µg/mL	11 (36.7)	7 (31.8)	.717	1.241 (0.387‐3.976)
Resistance of CLI	30 (96.8)	0 (0.0)	**<.001**	
Resistance of ERY	31 (100.0)	6 (27.3)	**<.001**	**5.429 (2.780‐10.599)**
Resistance of FUS	29 (93.5)	1 (4.5)	**<.001**	**304.500 (25.878‐3582.964)**
Resistance of GEN	21 (67.7)	3 (13.6)	**<.001**	**13.300 (3.177‐55.671)**
Resistance of RIF	10 (32.3)	0 (0.0)	**.003**	
Resistance of PEN	31 (100.0)	21 (95.5)	.415	
Resistance of TC	30 (96.8)	2 (9.1)	**<.001**	**300.000 (25.471‐3533.402)**
Virulence genes				
N. of detected virulence genes	10.3 ± 0.7	8.2 ± 0.7	**<.001**	
*Sea*	3 (9.7)	1 (4.5)	.633	2.250 (0.218‐23.191)
*sec*	21 (67.7)	0 (0.0)	<.001	
*sed*	6 (19.4)	0 (0.0)	.035	
*see*	1 (3.2)	0 (0.0)	1.000	
*seg*	29 (93.5)	22 (100.0)	.505	
*seh*	3 (9.7)	3 (13.6)	.683	0.679 (0.124‐3.726)
*sej*	2 (6.5)	1 (4.5)	1.000	1.448 (0.123‐17.041)
*sek*	2 (6.5)	2 (9.1)	1.000	0.690 (0.090‐5.310)
*sel*	26 (83.9)	5 (22.7)	**<.001**	**17.680 (4.438‐70.427)**
*sem*	28 (90.3)	19 (86.4)	.683	1.474 (0.268‐8.091)
*sen*	31 (100.0)	22 (100.0)		
*seo*	31 (100.0)	21 (95.5)	.415	
*sep*	5 (16.1)	0 (0.0)	.068	
*lukDE*	30 (96.8)	21 (95.5)	1.000	1.429 (0.085‐24.144)
*hlg*	1 (3.2)	0 (0.0)	1.000	
*fnbA*	4 (12.9)	0 (0.0)	.132	
*sdrE*	30 (96.8)	21 (95.5)	1.000	1.429 (0.085‐24.144)
30‐d mortality	14 (45.2)	6 (18.2)	.186	

Bold values indicate *P* <.05.

Abbreviations: CLI, clindamycin; ERY, erythromycin; FUS, fusidic acid; GEN, gentamicin; MIC, minimum inhibitory concentration; N., number; OR, odds ratio; PEN, penicillin; RIF, rifampicin; SD, standard deviation; TC, tetracycline.

The *sel* genes were more frequently detected in ST5 than in ST72 (OR 17.680 [4.438‐70.427], *P* < .001). The genes *sec* (*P* < .001) and *sed* (*P* = .035) were detected in ST5 but not in ST72.

We failed to observe any significant differences in the antibiotic susceptibility results of vancomycin MIC and the retained virulence genes between PFGE groups classified within the same STs.

All six ST72‐PFGE group A isolates had the same virulence genes (*seg*, *sei*, *sem*, *sen*, *seo*, *lukDE*, *sdrD*, and *sd*rE). Some ST72‐PFGE group B isolates harbored *sek*, *seh*, and *sel* aside from the virulence genes detected in PFGE group A. However, overall, no significant difference was observed in the virulence genes harbored and antibiotic susceptibility results involving vancomycin MIC between the PFGE groups classified in the same STs.

Twenty‐nine isolates of ST5, except the two isolates that were involved in PFGE group C and F, were clustered in PFGE group E. ST5 isolates showed no significant difference in antibiotic susceptibility results and virulence genes detected according to PFGE groups. In comparison to the other PFGE group E isolates, two ST632 isolates involved in PFGE group E were less likely to be resistant to antibiotics but showed no significant difference in virulence genes.

We assessed the 30‐day mortality rate in 57 patients with MRSAB strains, excluding two patients that could not be followed up because of transfer or discharge within 30 days of admission (detailed data are not shown). The 30‐day mortality rate was 38.6% (22/57) among patients with MRSAB. However, in our analysis, phenotypic and molecular factors were not related to outcome.

## 4 DISCUSSION

In this prospective study, we identified ST5 and ST72 as the major strains of MRSA involved in causing bacteremia. Previous studies have reported various ST strains for each region. In North America, CA‐MRSA, defined as USA300, was reported as ST8.^32^ In Western Europe, PVL‐positive strains, including ST80, were common.^33^ In Japan, ST5/ST764 are known as major HA‐MRSA.^17^ ST5 and ST72 have been reported to be the major HA‐ and CA‐MRSA in South Korea, and ST72 MRSA was widespread in community and hospital.^34^, ^35^, ^36^ Our results confirmed these results. We found that 86.4% of ST72 were HA‐MRSA, and the ratio of ST72 to entire isolates (37.3%) was higher than that reported in a previous study (22.4%).^15^ These results indicate that ST72 has already emerged as a major strain in hospital environment.

Even with the high discriminatory power of PFGE, the isolates belonging to the same ST were divided into only a few PFGE groups. We suggest that the bacteremia‐causing ST5 and ST72 strains of MRSA may be endemic without any new influx.

We observed significant differences in the antibiotic resistance patterns and virulence genes harbored between STs, especially ST5 and ST72. ST5 had more virulence genes and higher resistance rates against antibiotics than ST72. The *sel* genes were more frequently detected in ST5 than in ST72, and *sec* and *sed* were found only in ST5. The genes *sec* and *sel* were reported to be associated with ST5 in a previous report.^15^ These staphylococcal superantigen genes are known to play a critical role in the progression of *S aureus* infection.^37^ Therefore, ST5 strains carrying more staphylococcal superantigens may be highly virulent.^15^

We analyzed the mortality difference between ST5 and ST72 and failed to determine any statistical significance. However, the number of patients that died within 30 days was higher in ST5 group than in ST72 group. A previous study also reported lower mortality for ST72 than for ST5.^7^, ^15^

This study has some limitations. First, the exclusion of many patients may result in a bias analysis. Second, the number of isolates was insufficient to obtain statistical significance. Third, additional SCCmec typing needs to be carried out to identify whether ST5 and ST72 strains correspond to ST5‐SCCmecII and ST72‐SCCmecIV, which are established as the dominant strains of HA‐ and CA‐MRSA in South Korea. Fourth, other strain‐specific virulence genes that we failed to examine could exist and may play an important role in virulence. Therefore, further experiments involving whole‐genome studies should be performed to confirm the role of the virulence factors associated with the pathogenicity of MRSAB.

In conclusion, most MRSA isolates causing bacteremia were ST5 (CC5) and ST72 (CC8), and those belonging to the same STs were divided into only a few PFGE groups. The higher antibiotic resistance rate and staphylococcal superantigen toxin genes (*sec*, *sed*, and *sel*) in ST5 than in ST72 may be associated with its higher virulence capacity.
